# Incidents and Sudden Patient Deteriorations Occurring During Their Rehabilitation Sessions in an Acute Care Hospital: A Retrospective Cohort Study

**DOI:** 10.1016/j.arrct.2023.100307

**Published:** 2023-10-28

**Authors:** Koji Mizutani, Yohei Otaka, Masaki Kato, Miwako Hayakawa, Yoshitaka Wada, Takamichi Tohyama, Megumi Ozeki, Hirofumi Maeda, Satoshi Hirano, Seiko Shibata

**Affiliations:** a Department of Rehabilitation, Fujita Health University Hospital, Aichi, Japan; b Department of Rehabilitation Medicine I, Fujita Health University School of Medicine, Aichi, Japan; c Faculty of Rehabilitation, Fujita Health University, School of Health Sciences, Aichi, Japan

**Keywords:** Accidents, Complications, International Classification of Diseases, Rehabilitation, Risk management

## Abstract

**Objective:**

To investigate the occurrence of incidents and sudden deteriorations during rehabilitation in an acute care setting by disease category based on the International Classification of Diseases and Related Health Problems, 10th Revision.

**Design:**

Retrospective cohort study.

**Setting:**

University hospital in Japan with 1376 beds.

**Participants:**

A total of 49,927 patients who were admitted to the acute care wards and underwent rehabilitation over 8 years, from April 1, 2013, to March 31, 2021.

**Interventions:**

Rehabilitation in an acute care setting.

**Main Outcome Measures:**

Incidents and sudden deteriorations reported in medical charts.

**Results:**

Among 49,927 admissions, 455 incidents and 683 sudden deteriorations occurred during rehabilitation. The incidents and sudden deteriorations occurred at rates of 0.009/person (0.50 case/1000 h) and 0.012/person (0.75 case/1000 h), respectively. The 3 most frequent incidents were “route-related incidents” (178 cases, 39.1%), followed by “bleeding/abrasions” (131 cases, 28.8%) and “falls” (125 cases, 27.5%). Among 12 disease categories with over 500 admissions and 10,000 rehabilitation hours, the highest incident rate occurred in “certain infectious and parasitic diseases” (0.81 case/1000 h), followed by “diseases of the musculoskeletal system and connective tissue” (0.67 case/1000 h) and “diseases of the genitourinary system” (0.66 case/1000 h). The commonest sudden deterioration was “vomiting” (460 cases, 67.3%), followed by “decreased level of consciousness (with reduced blood pressure)” (42 cases, 6.1%) and “seizure” (39 cases, 5.7%). Furthermore, the highest sudden deterioration rate was in the “endocrine, nutritional, and metabolic diseases” (1.19 case/1000 h) category, followed by “neoplasms” (1.04 case/1000 h) and “certain infectious and parasitic diseases” (0.99 case/1000 h).

**Conclusions:**

An incident and sudden deterioration occurred every 2000 and 1333 h, respectively, during rehabilitation. Therefore, understanding the actual occurrence of incidents and sudden deteriorations during rehabilitation may provide valuable insights into preventing incidents and emergencies.

Medical personnel involved in patient care are responsible for safely treating patients and ensuring high-quality medical care. The Global Patient Safety Action Plan 2021-2030, recommended by the World Health Organization, envisions “a world in which no one is harmed in health care, and every patient receives safe and respectful care, every time, everywhere.”^1^

The effectiveness of rehabilitation in acute care, such as in intensive care units and the early stages after disease onset, is widely recognized.^2^, ^3^, ^4^, ^5^, ^6^ Rehabilitation is frequently initiated at disease onset or during the early postoperative period despite a high risk of sudden deterioration in patient condition. Many patients requiring rehabilitation also have impaired physical function or movement ability. Furthermore, as global aging continues, as observed in Japan,^7^^,^^8^ the prevalence of overlapping diseases is increasing,^9^ even as hospital stay for acute care is progressively decreasing yearly.^10^^,^^11^ Therefore, rehabilitation in acute care settings is associated with high risks of incidents and sudden deteriorations in patient condition. A trade-off exists between increased activity and safety,^12^ which emphasizes the need to prevent incidents during rehabilitation in the acute care and rehabilitation hospitals. Although the risk of sudden deteriorations and incidents is high in acute rehabilitation settings, only a few studies have analyzed this risk. Therefore, to predict and prevent medical incidents and sudden deteriorations in patient condition and provide safe rehabilitation, understanding the sudden deteriorations and incidents that can occur during rehabilitation sessions is important. We conducted a retrospective survey of the incidents and sudden deteriorations occurring during rehabilitation sessions at a university hospital.

## Methods

### Study setting

This study was conducted at the university hospital with the largest number of beds (1376) in Japan. This hospital is mainly responsible for acute care and has 11 acute care units (126 beds), including intensive, stroke, and coronary care units. Additionally, the hospital provides specialized intensive care based on disease type and severity, with a 60-bed ward for intensive rehabilitation.

Patients in all departments have access to rehabilitation services performed by therapists as prescribed by physicians. Between 2013 and 2021, an average of 13.3 rehabilitation physicians and 107.5 therapists (58.5 physical, 33.9 occupational, and 15.1 speech-language-hearing therapists) provided acute rehabilitation services.

Rehabilitation in acute-care hospitals in Japan is provided under the medical insurance system established by the Ministry of Health, Labor, and Welfare on an individual basis, with 1 therapist for each patient per time. The maximum duration of rehabilitation is 6-9 units (unit=20 min, 2-3 h in total) per patient daily, depending on the patient's disease and the disease phase. Additionally, the allowed period of rehabilitation (90-180 days) is determined based on the disease.

### Participants

This study's inclusion criteria were consecutive patients admitted to the hospital's acute care wards and underwent rehabilitation between April 1, 2013, and March 31, 2021. Patients who were admitted 2 or more times were also included. The exclusion criterion was patients who underwent rehabilitation in the rehabilitation ward. The local Medical Research Ethics Review Committee approved the study protocol. Informed consent was waived because of the study's retrospective design, and consenting patients were enrolled. This study followed STROBE guidelines.

### Data collection

Data on patients’ age; sex; length of hospitalization; disease classification according to the International Statistical Classification of Disease and Related Health Problems, 10th Revision (ICD-10); and place, time, and duration of rehabilitation were collected from medical records. When a patient was hospitalized 2 or more times, each hospitalization was treated as a single data set because incidents and sudden deteriorations might occur during each hospitalization. Details of incidents and sudden deteriorations in patient condition that occurred during rehabilitation, including the place and time of occurrence, were collected from medical records and the rehabilitation database. In this context, “incident” refers to “any unplanned or unintended event or circumstance caused by medical personnel, equipment, or systems within the medical facility that occurred during rehabilitation and could have resulted or resulted in harm to the patient” (eg, route-related incidents such as accidental intravenous line removal). Incidents were classified according to the 7 levels of effect on patients set by Japan Community Health Care Organization for Medical Safety Management^13^ (supplemental table S1). “Sudden deterioration” was defined as “a temporary interruption or discontinuation of rehabilitation due to a sudden deterioration in the patient's physical condition” (eg, vomiting and decreased level of consciousness). In our hospital, the therapist responsible for a patient's rehabilitation records incidents or sudden deteriorations in the rehabilitation database using a report form. These written reports are assessed by a senior therapist and the medical safety manager of the rehabilitation department to verify their accuracy and adequacy.

### Statistical analysis

The following descriptive statistical analyses were performed. The patient's demographic information and the number of incidents and sudden deteriorations that occurred during rehabilitation were investigated across 23 disease categories. These categories were adopted from the ICD-10 system, which includes 22 general disease categories. However, “cerebrovascular diseases” was considered a separate subcategory, distinct from the broader “diseases of the circulatory system.” This separation was due to the significant differences in rehabilitation between cardiovascular and cerebrovascular diseases.

## Results

During the study period, 49,927 admissions (34,916 unique patients) met the criteria. Overall, 8524 patients were admitted to 2 or more times. The mean age (SD) of the admissions, length of hospital stay, and duration of rehabilitation were 68.8 (18.1) years, 33.2 (35.6) days, and 25.5 (31.9) days, respectively. Overall, 1417,168 rehabilitation sessions were performed, including 1039,883 (73.4%) performed at the bedside. The disease category with the highest number of patients who underwent rehabilitation was “neoplasms” (12,124 patients) (table 1). Details of the rehabilitation provided for each disease category are shown in supplemental table S2.

**Table 1:** Characteristics of admissions who underwent rehabilitation according to disease category

ICD-10	n	Age	Men (%)	Length of hospital stay, days	Length of rehabilitation, days
Certain infectious and parasitic diseases	999	72.5 (15.6)	569 (57.0)	39.7 (41.7)	33.2 (40.8)
Neoplasms	12,124	68.2 (14.8)	6612 (54.5)	38.9 (36.1)	28.5 (31.2)
Diseases of the blood and blood-forming organs and certain disorders involving the immune mechanism	330	67.1 (23.1)	147 (44.5)	46.0 (78.4)	36.5 (77.3)
Endocrine, nutritional, and metabolic diseases	1403	68.3 (19.1)	672 (47.9)	28.6 (35.3)	21.4 (24.8)
Mental and behavioral disorders	634	59.3 (20.1)	200 (31.5)	57.0 (44.1)	46.0 (39.0)
Diseases of the nervous system	3854	59.6 (20.9)	1963 (50.9)	23.9 (33.7)	19.4 (32.3)
Diseases of the eye and adnexa	195	73.2 (13.4)	84 (43.1)	27.0 (28.7)	15.2 (23.9)
Diseases of the ear and mastoid process	52	76.3 (12.5)	20 (38.5)	13.6 (6.7)	8.1 (6.4)
Diseases of the circulatory system	10,507	72.6 (13.8)	6164 (58.7)	29.7 (28.1)	23.9 (25.9)
Diseases of the circulatory system (excluding cerebrovascular diseases)	4847	74.8 (13.0)	2805 (57.9)	32.8 (29.0)	24.0 (26.2)
Cerebrovascular diseases	5660	70.6 (14.1)	3359 (59.3)	27.0 (27.0)	23.8 (25.7)
Diseases of the respiratory system	3789	75.4 (16.2)	2590 (68.4)	26.8 (22.8)	21.0 (21.9)
Diseases of the digestive system	2302	74.3 (16.4)	1221 (53.0)	33.4 (34.5)	24.7 (30.2)
Diseases of the skin and subcutaneous tissue	418	71.0 (16.0)	197 (47.1)	49.5 (75.9)	35.8 (48.9)
Diseases of the musculoskeletal system and connective tissue	5284	67.0 (14.7)	2112 (40.0)	31.8 (34.3)	23.4 (30.4)
Diseases of the genitourinary system	2084	75.9 (13.5)	1133 (54.4)	32.0 (35.1)	24.6 (33.9)
Pregnancy, childbirth, and the puerperium	31	35.7 (8.5)	0 (0.0)	59.1 (56.0)	19.8 (28.5)
Certain conditions originating in the perinatal period	306	0.05 (0.5)	160 (52.3)	81.4 (78.3)	70.1 (73.9)
Congenital malformations, deformations, and chromosomal abnormalities	269	18.7 (24.5)	132 (49.1)	52.3 (57.7)	42.1 (54.0)
Symptoms, signs, and abnormal clinical and laboratory findings, not elsewhere classified	121	72.0 (19.4)	69 (57.0)	21.9 (22.4)	16.5 (21.7)
Injury, poisoning, and certain other consequences of external causes	5172	67.8 (20.7)	2480 (48.0)	32.1 (35.8)	25.5 (34.3)
External causes of morbidity and mortality	0				
Factors influencing health status and contact with health services	25	52.6 (17.6)	15 (60.0)	31.6 (30.9)	24.1 (29.8)
Codes for special purposes	28	71.2 (11.0)	19 (67.9)	40.2 (18.9)	18.0 (15.5)

NOTE. Values are presented as mean (SD) or number (%).

**Fig 1:**
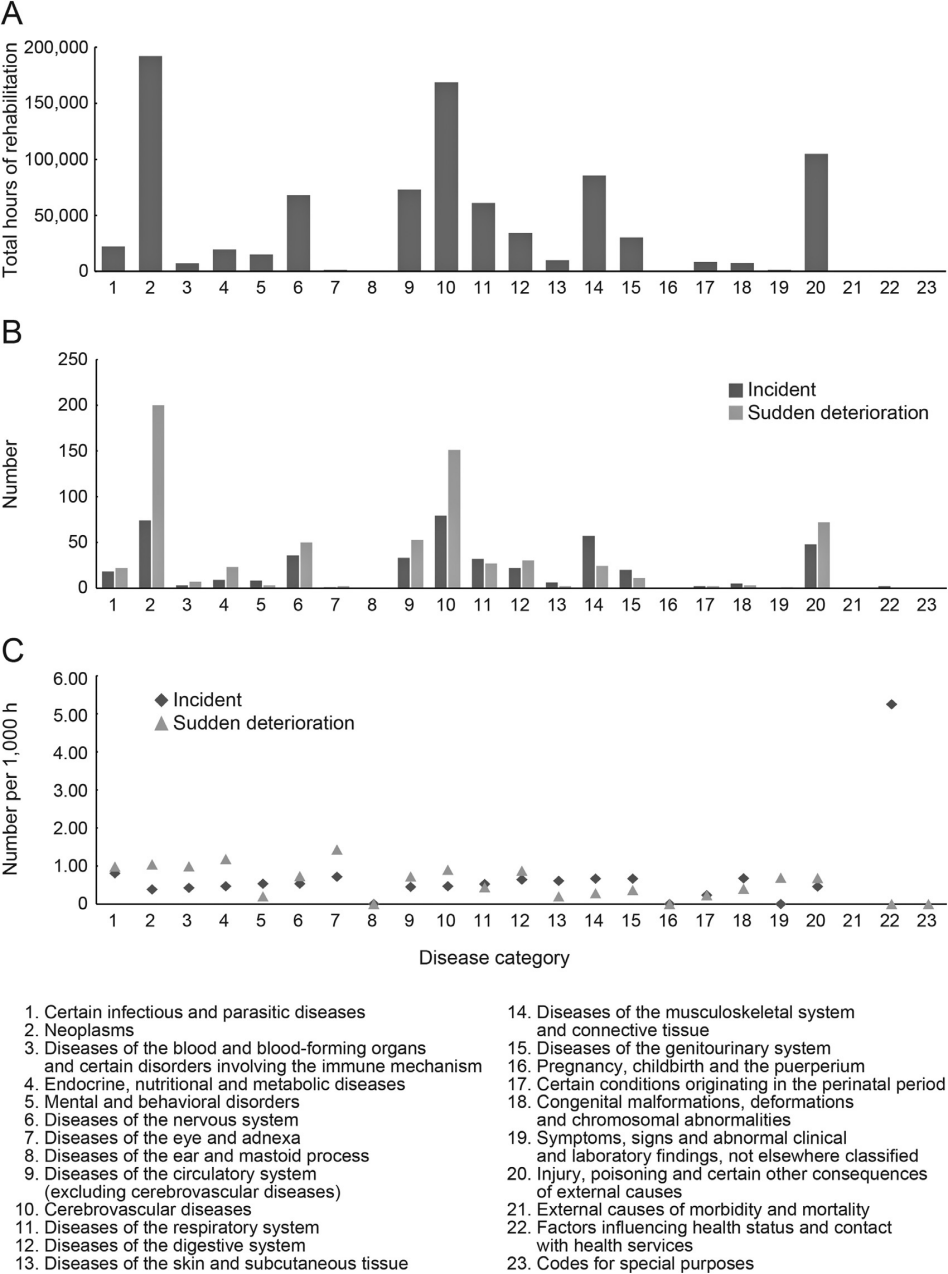
Amount of rehabilitation and rates of incidents and sudden deteriorations during rehabilitation sessions. Total hours of rehabilitation (A), numbers of incidents and sudden deteriorations (B), and their rates (C) are shown in each disease category.

Overall, 455 incidents occurred during the study period (fig 1 and supplemental table S3). The mean (SD) age of admissions who had incidents was 65.6 (20.0) years. The incident rates (number of incidents/number of admissions who underwent rehabilitation) by age category were 0.014/person (19 cases) for admissions <18, 0.010/person (135 cases) for those between 18 and 65, 0.010/person (127 cases) for those between 65 and 75, and 0.008/person (174 cases) for those >75. Nineteen admissions had multiple incidents during 1 hospitalization (18 had 2 incidents, and 1 had 3 incidents). The mean annual number of incidents per therapist was 0.53, and the commonest type of incident was “route-related incidents” (178 cases, 39.1%) (table 2). Effects of levels 1, 2, 3a, and 3b were reported in 268 (58.9%), 18 (4.0%), 165 (36.3%), and 4 ([0.9%] 3 cases of fracture and 1 of suture laceration caused by a fall) cases, respectively. Wards were the commonest locations for incidents (301 [66.2%]), compared with rehabilitation gyms and outdoors (148 [32.5%] and 6 [1.3%], respectively). The most frequent time of incident occurrence was 11:00 AM (15.8%, 72 cases), followed by 10:00 AM (14.1%, 64 cases), and 9:00 AM (12.5%, 57 cases), with no time having a particularly high incident occurrence rate.

**Table 2:** Details of incidents in each disease category

ICD-10	Route-related incidents	Bleeding/Abrasions	Falls	Pain	Fracture (non-fall related)	Others
Certain infectious and parasitic diseases	8 (1.8)	6 (1.3)	4 (0.9)			
Neoplasms	33 (7.3)	14 (3.1)	24 (5.3)	1 (0.2)	1 (0.2)	1 (0.2)
Diseases of the blood and blood-forming organs and certain disorders involving the immune mechanism	1 (0.2)	1 (0.2)	1 (0.2)			
Endocrine, nutritional, and metabolic diseases	1 (0.2)	3 (0.7)	5 (1.1)			
Mental and behavioral disorders	1 (0.2)	1 (0.2)	6 (1.3)			
Diseases of the nervous system	13 (2.9)	9 (2.0)	12 (2.6)	1 (0.2)		1 (0.2)
Diseases of the eye and adnexa	1 (0.2)					
Diseases of the ear and mastoid process						
Diseases of the circulatory system	51 (11.2)	29 (6.4)	27 (5.9)	1 (0.2)		4 (0.9)
Diseases of the circulatory system (excluding cerebrovascular diseases)	12 (2.6)	9 (2.0)	10 (2.2)			2 (0.4)
Cerebrovascular diseases	39 (8.6)	20 (4.4)	17 (3.7)	1 (0.2)		2 (0.4)
Diseases of the respiratory system	17 (3.7)	9 (2.0)	4 (0.9)	1 (0.2)		1 (0.2)
Diseases of the digestive system	10 (2.2)	7 (1.5)	5 (1.1)			
Diseases of the skin and subcutaneous tissue	1 (0.2)	4 (0.9)	1 (0.2)			
Diseases of the musculoskeletal system and connective tissue	13 (2.9)	21 (4.6)	20 (4.4)	3 (0.7)		
Diseases of the genitourinary system	5 (1.1)	7 (1.5)	7 (1.5)			1 (0.2)
Pregnancy, childbirth, and the puerperium						
Certain conditions originating in the perinatal period	2 (0.4)					
Congenital malformations, deformations, and chromosomal abnormalities	2 (0.4)	1 (0.2)	2 (0.4)			
Symptoms, signs, and abnormal clinical and laboratory findings, not elsewhere classified						
Injury, poisoning, and certain other consequences of external causes	19 (4.2)	17 (3.7)	7 (1.5)	1 (0.2)	2 (0.4)	2 (0.4)
External causes of morbidity and mortality						
Factors influencing health status and contact with health services		2 (0.4)				
Codes for special purposes						
Total	178 (39.1)	131 (28.8)	125 (27.5)	8 (1.8)	3 (0.7)	10 (2.2)

NOTE. Values are presented as numbers (percentages).

The disease category with the highest number of incidents was “cerebrovascular diseases” (79 cases), followed by “neoplasms” (74 cases) and “diseases of the musculoskeletal system and connective tissue” (57 cases). The disease category with the highest incidence of route-related incidents was “cerebrovascular diseases” (39 cases), followed by “neoplasms” (33 cases) and “injuries, poisoning, and certain other consequences of external causes” (19 cases). Additionally, the disease category with the highest incidence of bleeding/abrasions was “diseases of the musculoskeletal system and connective tissue” (21 cases), followed by “cerebrovascular diseases” (20 cases) and “injuries, poisoning, and certain other consequences of external causes” (17 cases). The disease category with the highest incidence of falls was “neoplasms” (24 cases), followed by “diseases of the musculoskeletal system and connective tissue” (20 cases) and “cerebrovascular diseases” (17 cases).

Overall, the incident rate per 1000 h of rehabilitation was 0.50. For the disease categories where a small amount of rehabilitation was provided, the incident rate was very high, even in the presence of a few events. Therefore, we separately analyzed 12 disease categories with more than 500 admissions and 10,000 h of rehabilitation. Among these categories, the one with the highest incident rate was “certain infectious and parasitic diseases” (0.81), followed by “diseases of the musculoskeletal system and connective tissue” (0.67) and “diseases of the genitourinary system” (0.66) (fig 1 and supplemental table S3).

In total, 683 cases of sudden deterioration were observed (fig 1 and supplemental table S3). The mean (SD) age of admissions with sudden deteriorations was 65.8 (20.1) years. Additionally, the rates of sudden deterioration (number of sudden deteriorations/number of admissions who underwent rehabilitation) by age category was 0.016/person (22 cases) for admissions <18, 0.017/person (216 cases) for those between 18 and 65, 0.011/person (147 cases) for those between 65 and 75, and 0.013/person (298 cases) for those >75. Sixty-one admissions had multiple sudden deteriorations during 1 hospitalization (45, 12, 2, 1, and 1 patients had 2, 3, 4, 5, and 9, respectively). The annual number of sudden deteriorations per therapist was 0.79. “Vomiting” was the commonest sudden deterioration (460 cases, 67.3%), followed by “decreased level of consciousness (with decreased blood pressure)” (42 cases, 6.1%) and “seizure” (39 cases, 5.7%) (table 3). Sudden deteriorations occurred in 528 (77.3%) and 155 (22.7%) patients in wards and rehabilitation gyms, respectively. The commonest time of sudden deterioration occurrence was 10:00 AM (16.3%, 111 cases), followed by 3:00 PM (15.4%, 105 cases) and 11:00 AM (15.2%, 104 cases), with no time having a particularly high sudden deterioration rate.

**Table 3:** Details of sudden deteriorations in each disease category

ICD-10	Vomiting	Decreased level of consciousness (with decreased blood pressure)	Seizure	Respiratory deterioration	Decreased level of consciousness (without decreased blood pressure)	Blood pressure changes (without a decrease in the level of consciousness)	Nausea/Discomfort Feeling	Arrhythmia	Cardiac arrest	Bleeding	Chest pain	Others
Certain infectious and parasitic diseases	10 (1.5)	1 (0.1)	2 (0.3)	4 (0.6)	1 (0.1)	3 (0.4)			1 (0.1)			
Neoplasms	144 (21.1)	12 (1.8)	13 (1.9)	11 (1.6)	5 (0.7)	7 (1.0)	1 (0.1)	1 (0.1)	1 (0.1)	3 (0.4)	1 (0.1)	1 (0.1)
Diseases of the blood and blood-forming organs and certain disorders involving the immune mechanism	3 (0.4)			1 (0.1)		2 (0.3)				1 (0.1)		
Endocrine, nutritional, and metabolic diseases	12 (1.8)	3 (0.4)	2 (0.3)	2 (0.3)	3 (0.4)		1 (0.1)					
Mental and behavioral disorders	2 (0.3)	1 (0.1)										
Diseases of the nervous system	29 (4.2)	3 (0.4)	6 (0.9)	4 (0.6)	4 (0.6)	2 (0.3)		1 (0.1)				1 (0.1)
Diseases of the eye and adnexa						2 (0.3)						
Diseases of the ear and mastoid process												
Diseases of the circulatory system	156 (22.8)	10 (1.5)	9 (1.3)	3 (0.4)	9 (1.3)	4 (0.6)	4 (0.6)	3 (0.4)	2 (0.3)		3 (0.4)	1 (0.1)
Diseases of the circulatory system (excluding cerebrovascular diseases)	40 (5.9)		1 (0.1)	1 (0.1)	2 (0.3)	1 (0.1)	1 (0.1)	3 (0.4)	1 (0.1)		2 (0.3)	1 (0.1)
Cerebrovascular diseases	116 (17.0)	10 (1.5)	8 (1.2)	2 (0.3)	7 (1.0)	3 (0.4)	3 (0.4)		1 (0.1)		1 (0.1)	
Diseases of the respiratory system	16 (2.3)		1 (0.1)	3 (0.4)	1 (0.1)	1 (0.1)	2 (0.3)		2 (0.3)	1 (0.1)		
Diseases of the digestive system	20 (2.9)	1 (0.1)	1 (0.1)	2 (0.3)	3 (0.4)	1 (0.1)		1 (0.1)		1 (0.1)		
Diseases of the skin and subcutaneous tissue	1 (0.1)				1 (0.1)							
Diseases of the musculoskeletal system and connective tissue	11 (1.6)		1 (0.1)	1 (0.1)	3 (0.4)	4 (0.6)	1 (0.1)	1 (0.1)		1 (0.1)	1 (0.1)	
Diseases of the genitourinary system	4 (0.6)	2 (0.3)	1 (0.1)	3 (0.4)				1 (0.1)				
Pregnancy, childbirth, and the puerperium												
Certain conditions originating in the perinatal period	2 (0.3)											
Congenital malformations, deformations, and chromosomal abnormalities	2 (0.3)			1 (0.1)								
Symptoms, signs, and abnormal clinical and laboratory findings, not elsewhere classified		1 (0.1)										
Injury, poisoning, and certain other consequences of external causes	48 (7.0)	8 (1.2)	3 (0.4)	2 (0.3)	3 (0.4)	3 (0.4)	2 (0.3)		1 (0.1)			2 (0.3)
External causes of morbidity and mortality												
Factors influencing health status and contact with health services												
Codes for special purposes												
Total	460 (67.3)	42 (6.1)	39 (5.7)	37 (5.4)	33 (4.8)	29 (4.2)	11 (1.6)	8 (1.2)	7 (1.0)	7 (1.0)	5 (0.7)	5 (0.7)

NOTE. Values are presented as numbers (percentages).

The disease category with the highest number of sudden deteriorations was “neoplasms” (200 cases), followed by “cerebrovascular diseases” (151 cases) and “injury, poisoning, and certain other consequences of external causes” (72 cases). “Neoplasms” were most commonly associated with vomiting (144 cases), followed by “cerebrovascular diseases” (116 cases) and “injury, poisoning, and certain other consequences of external causes” (48 cases). The disease category most commonly associated with a decreased level of consciousness (with decreased blood pressure) was “neoplasms” (12 cases), followed by “cerebrovascular diseases” (10 cases) and “injury, poisoning, and certain other consequences of external causes” (8 cases). Furthermore, the disease category with the highest incidence of seizures was “neoplasms” (13 cases), followed by “cerebrovascular diseases” (8 cases) and “diseases of the nervous system” (6 cases).

The rate of sudden deterioration per 1000 h of rehabilitation was 0.75. Similar to the analysis for incidents, we separately analyzed 12 disease categories with more than 500 admissions and 10,000 h of rehabilitation. Among the categories, “endocrine, nutritional, and metabolic diseases” (1.19) was the one with the highest rate of sudden deterioration, followed by “neoplasms” (1.04) and “certain infectious and parasitic diseases” (0.99) (fig 1 and supplemental table S3).

## Discussion

We investigated and clarified the current status of incidents and sudden deteriorations that occurred during acute rehabilitation according to the disease category. To our knowledge, this is the first report on the current status of incidents and sudden deteriorations that occur during rehabilitation in acute care settings by disease category.

In our analysis, “route-related incidents” were the most common incidents. In acute care hospitals, rehabilitation is usually initiated while the patient receives therapy through intravenous drips and other devices. During exercises involving physical activity, an increased risk of disconnecting a route connected to a patient exists when the therapist has inadequate control over it. Additionally, patients who are restless, inattentive, or uncomfortable with nasogastric tubes or other routes are likely to remove them during rehabilitation unintentionally. Therefore, “route-related incidents” were the most common incidents in our study. The incident with the second highest frequency was “bleeding/abrasions.” The ability to perform activities of daily living is low in many patients receiving acute care at the initiation of rehabilitation^14^; they usually require assistance for movement or training to walk with a lower limb orthosis. Therefore, these patients are prone to bleeding and abrasions from bumping into the bed rail or wheelchair when getting up from the bed and transferring into the wheelchair or using the lower limb orthosis during exercise. The third most frequent incident was “falls.” Patients receiving rehabilitation frequently require difficult exercises to acquire movement abilities. Although tasks of appropriate difficulty are essential to acquire movement skills, they increase the risk of falls. Of the 455 incidents that occurred during rehabilitation, 4 (0.9%) were of level 3b effect, requiring intensive treatment. No incidents that resulted in permanent disability or residual effects (level 4) or death (level 5) occurred, indicating the relative safety of rehabilitation.

The highest rates of incidents per 1000 h were found in “certain infectious and parasitic diseases” category, followed by “diseases of the musculoskeletal system and connective tissue” and “diseases of the genitourinary system.” The mean rate of incidents per 1000 h was 0.55 for the 12 disease categories, and the variation by disease category was small, suggesting that the number of incidents is independent of the disease but depends on the number of patients undergoing rehabilitation.

“Vomiting” was the commonest sudden deterioration, accounting for 67.3% of cases, mainly involving “neoplasms,” followed by “cerebrovascular diseases.” Many patients admitted to acute care facilities have an unstable general condition or are taking drugs with a vomiting side effect, such as anticancer drugs. It was reasonable that “vomiting” during rehabilitation was most common in “neoplasms,” followed by the disease category with the largest number of patients undergoing rehabilitation and conditions associated with vomiting. The second commonest sudden deterioration was “decreased level of consciousness (with reduced blood pressure).” Patients in acute care settings tend to be confined to the bed or have limited activity during treatment. Additionally, patients may have decreased circulating blood volumes immediately after hospitalization due to dehydration caused by decreased eating and drinking. In these situations, patients are prone to decreased consciousness and blood pressure levels because of reduced circulating blood volume caused by the inflow of blood to the extremities due to limb exercises, movement practice, and postural changes during rehabilitation. Furthermore, cerebrovascular disease may be associated with impaired blood pressure regulation and orthostatic hypotension, commonly occurring in the early stroke phase.^15^ The third commonest sudden deterioration was “seizure,” which predominantly involved patients with “neoplasms,” “cerebrovascular diseases,” and “diseases of the nervous system.” Seizures were likely to occur in patients with brain tumors and meningiomas, which are categorized as “neoplasms,” and in those with central nervous system diseases, categorized as “cerebrovascular diseases” and “diseases of the nervous system.” Therefore, “seizure” was considered to have occurred more frequently in these disease categories than in the others.

The incidence rates of sudden deterioration per 1000 h of rehabilitation were “endocrine, nutritional, and metabolic diseases” (1.19 cases), “neoplasms” (1.04 cases), and “certain infectious and parasitic diseases” (0.99 cases). The mean (SD) incidence rate of sudden deterioration per 1000 h was 0.70 (0.32) for the 12 disease categories with the largest numbers of patients and hours of rehabilitation, showing a variation in the incidence rate per disease category compared with incidents. The “endocrine, nutritional, and metabolic diseases” category includes patients with diabetes mellitus, who may be prone to hypoglycemia symptoms, such as vomiting and decreased consciousness level. Patients with “neoplasms” and “certain infectious and parasitic diseases” frequently use drugs with side effects, such as anticancer agents and antibiotics. Additionally, patients with “certain infectious and parasitic diseases” were more likely to have unstable vital signs, such as fever, dehydration, and blood pressure changes, than other patients. Therefore, these factors were responsible for the high incidence of sudden deteriorations.

### Study limitations

This study was conducted at a single facility in Japan, which may have introduced a sampling bias. Therefore, a multicenter, multinational survey is required to examine variations among facilities and nations that employ different medical systems.

## Conclusions

This study elucidated the incidents and sudden deteriorations that occurred during acute rehabilitation at a university hospital. Incident rates showed limited variations across the disease categories, and specific diseases did not significantly influence the rates. However, the sudden deterioration rate varied between the disease categories. Therefore, this variation is believed to have stemmed from the nature of the diseases and their treatments, which may have led to fluctuations in the patient's conditions.

## Appendix Supplementary materials

Image, application 1

Image, application 2

Image, application 3

## Footnotes

Supplementary material associated with this article can be found, in the online version, at doi:10.1016/j.arrct.2023.100307.
