# Supplementary material for: Incidents and Sudden Patient Deteriorations Occurring During Their Rehabilitation Sessions in an Acute Care Hospital: A Retrospective Cohort Study
Source: Arch Rehabil Res Clin Transl. 2023 Oct 28;5(4):100307. doi: 10.1016/j.arrct.2023.100307 (PMC10757191; doi:10.1016/j.arrct.2023.100307)
Supplement: Supplementary file 1 [file mmc1.docx]

Supplemental Table 1. Classification of impact on the patients

|  | Level | Continuity of injury | Severity of injury Contents |
| --- | --- | --- | --- |
|  | 0 |  | Errors and problems with medicines and medical equipment were observed, but not carried out on the patient. |
|  | 1 | No | No actual harm There was no harm to the patient (there was a possibility of some influence) |
|  | 2 | Transient | Mild No treatment or therapy was administered (minor changes in vital signs, increased observation, and safety checkups became necessary). |
|  | 3a | Transient | Moderate Required simple procedures or treatments (disinfection, compresses, skin suture, administration of  analgesics, etc.) |
|  | 3b | Transient | Severe Required intensive care or treatment (advanced change in vital signs, ventilator, surgery, extended hospital stay, hospitalization, fractures, etc.) |
|  | 4 | Permanent | Mild to severe Permanent disability or sequelae (with or without significant functional impairment or cosmetic problems) |
|  | 5 | Death | Death (excluding death due to the natural history of underlying disease) |
|  |  |  |  |
