# Supplementary material for: Incidents and Sudden Patient Deteriorations Occurring During Their Rehabilitation Sessions in an Acute Care Hospital: A Retrospective Cohort Study
Source: Arch Rehabil Res Clin Transl. 2023 Oct 28;5(4):100307. doi: 10.1016/j.arrct.2023.100307 (PMC10757191; doi:10.1016/j.arrct.2023.100307)
Supplement: Supplementary file 2 [file mmc2.docx]

Supplemental Table 2. Details of the rehabilitation provided according to the disease category

| ICD-10* | Total number of admissions who underwent rehabilitation | Number of admissions who underwent physical therapy | Number of admissions who underwent occupational therapy | Number of admissions who underwent speech-language-hearing therapy | Total time of rehabilitation, hours | Total time of physical therapy, hours | Total time of occupational therapy, hours | Total time of speech-language-hearing therapy, hours |
| --- | --- | --- | --- | --- | --- | --- | --- | --- |
| Certain infectious and parasitic diseases | 999 | 992 | 500 | 186 | 22,323 | 13,364 | 6,233 | 2,726 |
| Neoplasms | 12,124 | 11,464 | 6,410 | 1,332 | 192,110 | 108,543 | 63,897 | 19,670 |
| Diseases of the blood and blood-forming organs and certain disorders involving the immune mechanism | 330 | 329 | 163 | 36 | 7,041 | 4,277 | 1,919 | 845 |
| Endocrine, nutritional and metabolic diseases | 1,403 | 1,392 | 661 | 152 | 19,407 | 11,953 | 5,727 | 1,728 |
| Mental and behavioral disorders | 634 | 584 | 299 | 63 | 15,133 | 9,475 | 4,701 | 957 |
| Diseases of the nervous system | 3,854 | 3,772 | 3,054 | 1,147 | 67,916 | 30,610 | 26,021 | 11,285 |
| Diseases of the eye and adnexa | 195 | 189 | 94 | 9 | 1,396 | 912 | 445 | 39 |
| Diseases of the ear and mastoid process | 52 | 48 | 26 | 0 | 247 | 143 | 104 | 0 |
| Diseases of the circulatory system | 10,507 | 10,446 | 7,347 | 3,533 | 241,504 | 112,492 | 85,345 | 43,666 |
| Diseases of the circulatory system (excluding cerebrovascular diseases) | 4,847 | 4,822 | 1,805 | 519 | 72,934 | 48,150 | 17,799 | 6,985 |
| Cerebrovascular diseases | 5,660 | 5,624 | 5,542 | 3,014 | 168,570 | 64,342 | 67,546 | 36,681 |
| Diseases of the respiratory system | 3,789 | 3,772 | 1,990 | 886 | 60,775 | 32,289 | 18,428 | 10,058 |
| Diseases of the digestive system | 2,302 | 2,293 | 1,044 | 235 | 34,198 | 21,822 | 9,195 | 3,182 |
| Diseases of the skin and subcutaneous tissue | 418 | 411 | 237 | 46 | 9,940 | 5,687 | 3,795 | 458 |
| Diseases of the musculoskeletal system and connective tissue | 5,284 | 5,169 | 2,829 | 174 | 85,469 | 51,769 | 30,334 | 3,365 |
| Diseases of the genitourinary system | 2,084 | 2,074 | 1,064 | 223 | 30,214 | 17,827 | 9,779 | 2,607 |
| Pregnancy, childbirth and the puerperium | 31 | 31 | 7 | 0 | 309 | 221 | 88 | 0 |
| Certain conditions originating in the perinatal period | 306 | 306 | 31 | 27 | 8,496 | 6,618 | 962 | 916 |
| Congenital malformations, deformations and chromosomal abnormalities | 269 | 259 | 94 | 58 | 7,436 | 4,288 | 1,989 | 1,159 |
| Symptoms, signs and abnormal clinical and laboratory findings, not elsewhere classified | 121 | 118 | 66 | 21 | 1,442 | 796 | 464 | 182 |
| Injury, poisoning and certain other consequences of external causes | 5,172 | 5,020 | 3,229 | 588 | 104,844 | 57,393 | 38,264 | 9,187 |
| External causes of morbidity and mortality | 0 | 0 | 0 | 0 | 0 | 0 | 0 | 0 |
| Factors influencing health status and contact with health services | 25 | 25 | 7 | 2 | 381 | 244 | 84 | 53 |
| Codes for special purposes | 28 | 28 | 15 | 2 | 397 | 286 | 97 | 13 |
| Total | 49,927 | 48,722 | 29,167 | 8,720 | 910,978 | 491,010 | 307,872 | 112,096 |

Values are presented as numbers. *ICD-10, International Classification of Diseases and Related Health Problems, 10th Revision.
