# Supplementary material for: Incidents and Sudden Patient Deteriorations Occurring During Their Rehabilitation Sessions in an Acute Care Hospital: A Retrospective Cohort Study
Source: Arch Rehabil Res Clin Transl. 2023 Oct 28;5(4):100307. doi: 10.1016/j.arrct.2023.100307 (PMC10757191; doi:10.1016/j.arrct.2023.100307)
Supplement: Supplementary file 3 [file mmc3.docx]

Supplemental Table 3. Detailed data on the amount of rehabilitation and rates of incidents and sudden deteriorations during rehabilitation sessions in each disease category

| ICD-10* | Total time of rehabilitation, h | Number of incidents | Number of incidents per 1,000 h | Number of sudden deteriorations | Number of sudden deteriorations per 1,000 h |
| --- | --- | --- | --- | --- | --- |
| Certain infectious and parasitic diseases | 22,323 | 18 | 0.81 | 22 | 0.99 |
| Neoplasms | 192,110 | 74 | 0.39 | 200 | 1.04 |
| Diseases of the blood and blood-forming organs and certain disorders involving the immune mechanism | 7,041 | 3 | 0.43 | 7 | 0.99 |
| Endocrine, nutritional and metabolic diseases | 19,407 | 9 | 0.46 | 23 | 1.19 |
| Mental and behavioral disorders | 15,133 | 8 | 0.53 | 3 | 0.20 |
| Diseases of the nervous system | 67,916 | 36 | 0.53 | 50 | 0.74 |
| Diseases of the eye and adnexa | 1,396 | 1 | 0.72 | 2 | 1.43 |
| Diseases of the ear and mastoid process | 247 | 0 | 0.00 | 0 | 0.00 |
| Diseases of the circulatory system | 241,504 | 112 | 0.46 | 204 | 0.84 |
| Diseases of the circulatory system  (excluding cerebrovascular diseases) | 72,934 | 33 | 0.45 | 53 | 0.73 |
| Cerebrovascular diseases | 168,570 | 79 | 0.47 | 151 | 0.90 |
| Diseases of the respiratory system | 60,775 | 32 | 0.53 | 27 | 0.44 |
| Diseases of the digestive system | 34,198 | 22 | 0.64 | 30 | 0.88 |
| Diseases of the skin and subcutaneous tissue | 9,940 | 6 | 0.60 | 2 | 0.20 |
| Diseases of the musculoskeletal system and connective tissue | 85,469 | 57 | 0.67 | 24 | 0.28 |
| Diseases of the genitourinary system | 30,214 | 20 | 0.66 | 11 | 0.36 |
| Pregnancy, childbirth and the puerperium | 309 | 0 | 0.00 | 0 | 0.00 |
| Certain conditions originating in the perinatal period | 8,496 | 2 | 0.24 | 2 | 0.24 |
| Congenital malformations, deformations and chromosomal abnormalities | 7,436 | 5 | 0.67 | 3 | 0.40 |
| Symptoms, signs and abnormal clinical and laboratory findings, not elsewhere classified | 1,442 | 0 | 0.00 | 1 | 0.69 |
| Injury, poisoning and certain other consequences of external causes | 104,844 | 48 | 0.46 | 72 | 0.69 |
| External causes of morbidity and mortality |  |  |  |  |  |
| Factors influencing health status and contact with health services | 381 | 2 | 5.24 | 0 | 0.00 |
| Codes for special purposes | 397 | 0 | 0.00 | 0 | 0.00 |
| Total | 910,978 | 455 | 0.50 | 683 | 0.75 |

Values are presented as numbers. *ICD-10, International Classification of Diseases and Related Health Problems, 10th Revision
